# A Board of Medical Examiners: The State’s Medical Duty

**Published:** 1893-05

**Authors:** Luther B. Grandy

**Affiliations:** Atlanta, Ga.


ATLANTA
MEDICAL AND SURGICAL JOURNAL.

VOL. X. MAY, 1893. No. 3.

EDITED BY
LUTHER B. GRANDY, M. D., AND MILLER B. HUTCHINS, M. D.,
WITH THE CO-OPERATION OF
H. V. M. MILLER, M. D., LL. D., VIRGIL O. HARDON, M. D.,
FLOYD W. McRAE, M. D., AND ARTHUR G. HOBBS, M. D.

### A BOARD OF MEDICAL EXAMINERS: THE STATE'S MEDICAL DUTY.*

By LUTHER B. GRANDY, M. D.,
ATLANTA, GA.

In November last I suggested to the Atlanta Society of Medicine the advisability of taking steps toward the establishment of a Board of Medical Examiners in Georgia. The suggestion was acted upon with a promptness and enthusiasm which, I may say, both gratified and surprised me. A committee of prominent members of the Atlanta profession was appointed, and these gentlemen prepared a bill at once and submitted it to the legislature then in session. It passed the Senate by a vote of thirty-five to nine. From the beginning it encountered the rabid opposition of the Eclectics, Homeopaths, and one large member of the regular profession, toward whom, for personal reasons, I shall here indulge the charity of silence. Alarmed by the vote in the Senate, this triple but anomalous alliance sought to strengthen their unworthy cause by employing a learned counsel from Savannah who, for a consideration,

*Read at the meeting of the Georgia Medical Association, Americus, April 20, 1893.

doubtless, discovered that the bill was unconstitutional. Not only that, but there was another lawyer at the capitol hired to oppose this bill *by a notorious peripatetic quack doctor from Boston!* Such was the opposition. Such is the opposition to progressive and protective medical legislation everywhere. It is ever the same old story of the combination of quack doctors with those whose conception of medical teaching is a "college for revenue only." Before the Committee of the House the matter was thus brought to such inextricable confusion that further action was postponed until the next meeting of the legislature in the Fall. So that the bill may be considered as still pending.

It is not too much to say that this is a question which touches every home in Georgia, into which sickness or accident will surely come at one time or another. There are two classes of so-called doctors from whom the people of this and every State need to be protected. One is the ignorant practitioner without capacity, the other is the unprincipled charlatan without conscience. As I have already said in another place (Atlanta Medical and Surgical Journal, November, 1892), "a large proportion of the citizens of any State have not intelligence enough to protect themselves against these persons. Then it becomes the duty of the State to protect them. If those who offer their professional services to the community were everywhere what they should be, both in intelligence and integrity, there would be no cause for legislation. But the disagreeable fact remains that the medical schools of the country every year send out young men totally unqualified for practice, while there are those also so devoid of every sentiment of honor that they are willing to extract their dishonest livelihood by preying upon the ignorance and credulity of those whom they are able to deceive. Their brazen faces and lying advertisements disgrace the columns of the daily press, which will print anything for money. They cure the incurable, remove the unremovable, and accomplish the impossible, all either 'by consultation or by mail,' 'without the use of the knife,' with 'satisfaction guaranteed.' I am glad to say that the quack's field of operation is becoming more and more restricted year by year as the light dawns upon the intelligence of State legislatures. The door of quackery is closing in this country, and those who make their shameless living out of it are being forced to seek hospitable shelter in those States yet willing to harbor them. In Georgia, which boasts of being an Empire State, these Ishmaels in the profession find abundant opportunity still to ply their nefarious trade, with none to molest or make afraid."

I fancy that the English poet (Crabbe), with prophetic vision, must have been reading the pages of the Atlanta *Constitution* or the *Evening Journal* when he wrote, early in this century:

> . . . Our quacks are gamesters, and they play
> With craft and skill to ruin and betray;
> With monstrous promise they delude the mind,
> And thrive on all that tortures human-kind.
> And twenty names of cobblers turned to squires,
> Aid the bold language of these blushless liars;
> And then in many a paper through the year
> Must cures and cases, oaths and proofs appear.

It should touch our State pride, I think, that anybody can practice medicine in Georgia who has fifty cents to pay the county clerk. No diploma is necessary, and no license is furnished. But we must register our names in the court-house in order that the sheriff may know from whom he is to collect the professional tax of ten dollars per year. If one wishes to run a cab or dray he must get a license; if he wishes to sell meats, or drugs, or cigars, or soda water he must get a license; if he is a pawnbroker he must get a license; if he wishes to insure you or your property he must get a license; if he wishes to peddle wares at your door he must get a license; if he opens a theater to furnish you with recreation or amusement he must get a license; but one can physic you with aconite and strychnine, or open your abdomen, *without a license.*

The mistake which underlies the present law, if it may in charity be called a law, is in giving to a college diploma the authority of a license. This is indeed the *fons et origo mali.* It is giving too much power to colleges, actuated as they may be and often are, by personal interests, to make them the judges as to the qualifications of those who wish to practice medicine. The mission of the college is to *teach* the student; to *license* him to practice is quite a separate and distinct duty belonging to a separate and distinct power. The President of Harvard University has said: "Why is it that admission to the profession of medicine (in Massachusetts) is at a disadvantage as compared with admission to the profession of law? The very last thing the law school or the law faculty would desire would be that their degree should admit to the bar. It is a clear disadvantage in medical education that the degree given by a faculty, a teaching faculty, should admit to the profession. The standard should always be outside, determined by another power."

The United States is the only country in the world in which this blunder is allowed. A diploma is no guarantee of professional fitness. The value and significance of a bare diploma are diminished now by the commercial spirit which plays too important a rôle in our teaching institutions. Our law-makers may not know it, but it is a fact that there are too many teachers in our faculties everywhere who appear to believe that the success of their teaching plant is proportional to the amount of money that may be gotten out of it. It is this spirit which graduates students every year and declares them competent to practice a responsible profession who ought to be sawing wood or making brick.

A diploma a license! What a terrible mistake! How many cases of suffering and death can this audience relate, traceable to that dangerous paradox? Think I am exaggerating? I saw a child that had been treated for ten days with liniments for a supposed sprain of the hip-joint, resulting from a fall. The evidences of dorsal dislocation were so unmistakable that the wayfaring man need not have erred in the diagnosis. Under chloroform the dislocation was easily reduced, a large abscess followed which had to be opened, and the little patient suffered, from first to last, for many weeks. Why? Because the doctor first called didn't know his business. But he had a diploma, and a *diploma is a license in Georgia.* On this same subject a recent writer * says: "I stood by the body of a woman, and as the post mortem progressed men turned pale. There before us was a wound from vagina to abdominal cavity, through which the hand could be thrust, telling an awful story of shock, hemorrhage and death. The practitioner who made that wound had been trying to force a

*Dr. S. S. Towler, Marionville, Pa., *Medical and Surgical Reporter*, November 19, 1892.

pair of placental forceps into the os uteri, and had madly and ignorantly pushed the instrument through the cul de sac above." Thus a life was taken, and a whole family made unhappy through the criminal blunder of an ignorant physician. But he doubtless had a college diploma, *and in his State also a diploma is a license.*

The medical degree can be too easily and cheaply gotten in this country. Did you know that there are places where a diploma may be bought for a mere song, and no questions asked? A New York *Herald* reporter recently bought the title "Doctor of Medicine" from one of these for fifty dollars. There are two hundred and twenty-five "graduates" of these bogus institutions practicing medicine *upon their diplomas* in Pennsylvania. For this sort of "doctor," so-called, conceived in sin and born in iniquity, an honorable people and an honorable profession can have nothing but contempt. I do not know if there is any of this unlawfully-begotten species in Georgia; but there may be, *and his diploma is his license.*

The absence of such a board and the fact that a diploma is a license is but throwing down the bars to all the incompetent and fraudulent who are being rejected in other places. And here is the point of this matter which is of the most interest to us as physicians; because it has such a bearing upon the *personnel* of our State profession. If the medical profession in Georgia is to be made up largely of those who have been refused by other States on account of incompetency or dishonesty, then it is time we were entering our protest at once. There are about 2,700 physicians in Georgia, and still they come, and all are welcome. None are ever rejected. By a reasonable estimate about 700 of these would have been declared unqualified in other States. There have been nearly 400 rejections by the Virginia and North Carolina boards within the last seven years. What do you suppose becomes of all those persons who are examined and declined by the boards of New Jersey, Virginia, North Carolina, Florida, Alabama, Illinois, Minnesota and other States? Our neighbors, Alabama, South Carolina and Florida, have rejected respectively 20, 29 and 30 per cent. of their applicants. Where do these people go? As for the quacks, when driven from one place they quickly take refuge in another. They change their sky but not their affections (*Cœlum, non animum, mutant*). Like other moving bodies they travel along the lines of least resistance until they find some place still willing to receive and patronize them. The situation in Massachusetts to-day is proof of this statement. Dr. Draper, of Boston, says:

"One State after another has passed restrictive laws of greater or less stringency with the single aim of discouraging quackery. Massachusetts (and we will add Georgia) stands almost alone in her attitude of toleration. The action of neighboring States, near and more distant, in requiring irregular practitioners to move on and to stand not upon the order of their going, has brought to our too hospitable territory a horde of medical pretenders who have not been slow in discovering the advantages of an asylum here."

The experience of Illinois demonstrates how the *personnel* of the profession may be affected by such legislation as we now contemplate. In the thirteen years ending January 1st, 1891, the State Board of Health rejected 2,283 candidates for license. In 1877, when their law went into effect, there were 7,400 physicians in the State, 3,600 graduates and 3,800 non-graduates. Ten years later there were 6,065 physicians, of whom 5,327 were graduates, 738 non-graduates. In 1877 the percentage of graduates was 48; in 1886 it was 87. In 1877 the percentage of non-graduates was 52; in 1886 it was 13. In spite of large increase in population during the ten years there was a decrease of 1,335 in the number of physicians in the State, and a decrease of 3,062 in the number of non-graduates, with an increase of 1,727 in the number of graduate physicians.* All of which means that Illinois now has a capable, honest and intelligent profession, the result solely of efficient restrictive legislation.

One of the most important and salutary results of this sort of legislation is its effect upon medical education. The existence of so many of these examining boards in the States has been one of the most potent factors in arousing the colleges of the country to better work. The latter know and appreciate that the character and thoroughness of their teaching will be measured by the fitness or unfitness of their graduates as determined by

*Dr. W. L. McCreary, in *Southern Journal Homeopathy*, January, 1893.

these examinations. The consequence has been that our system of medical education has been overhauled and improved within the last five years as never before. In this respect the Southern colleges have not done their duty. There are about 140 medical schools in this country and Canada; 130 of these require a reasonable preliminary education before matriculation. How many of this number are in the South? Only three. Of the remaining ten which make no effort to ascertain if the applicant is capable of receiving a medical education, nine are Southern colleges. Of the seventy-eight American colleges which require three or more years of study and three terms of attendance upon lectures only six are in the South, and *three of these are schools for negroes.* There are not over fifteen colleges in this country which now graduate students at the end of their second course of lectures. Twelve of these are Southern colleges, and it is from these that Georgia gets her doctors, or the greater part of them. The science of medicine has made such progress within the last few years that it is no longer possible for a student to obtain a reliable medical education in two courses of study. This is a fact which no one will deny unless he be one of those who worships the commercial ideal in medical teaching. The schools of the North, for the most part, in spite of every facility of laboratory and hospital, require of their students a preliminary education and three or four terms of study and observation. But we of the South exact no previous preparation, and undertake in two ungraded courses of lectures of five months each to make doctors of those who come up from the farms. In medical education the proper solution lies in the application of the Malthusian principle: "Fewer conceptions and a prolonged period of gestation." As it is, we are having a premature delivery of young doctors every Spring. They come—

> Deformed, unfinished, sent before their time
> Into this (medical) world, scarce half made up, and there is nothing to stand between them and the people among whom they choose to locate. One of these men once said that the foramen magnum transmitted the arch of the aorta; another, that in post-partum hemorrhage he would ligate the post-partum artery; another, that for œdema of the glottis he would amputate in about two weeks; another, that rectocele was an inflammation of the cele of the rectum; another, that pyelitis was an inflammation of the pylorus; another said that the endothelium was a worm generally found in the rectum; and another, that in "hypertrophy of the heart we have a full cavity, while in dilatation we have a reservoir full of gas." In the hands of such persons the lives of the people are not safe, and there is properly no place for them in the medical profession; but some of these very ones, or their equivalents, are now practicing serenely in Georgia.

I am glad to say that an attempt has been made to correct this state of things. The Southern Medical College Association was organized in Louisville last Fall for the purpose of "elevating the standard of medical education by requiring a more thorough preliminary training and an increased length of medical study." Certain qualifications for matriculation were agreed upon and the three-term course adopted. But now what do we see? Every Southern college, except one, entered cheerfully into the spirit of this Association, anxious to place the status of medical education upon a higher plane in the South. The only one that refused is a Georgia college (the *Atlanta Medical College*), and to it now belongs the inglorious distinction of being probably the only school in this country that insists upon adhering to the old system. The refusal of one college may nullify the desires of all the rest. If so, the cause of higher medical education must languish in the South; the two-term system must prevail; and the colleges must continue to confer diplomas without giving an education; all because "Ephraim is joined to his idols" in Atlanta.

Every possible objection is going to be urged against the passage of this bill by those whose interest it is to oppose it. The regular, irregular, quack and legal affinities above alluded to will labor with one accord for its defeat. Our esteemed eclectic cousins have on their war paint and are already in the ring. The clans gathered in February last and selected a "strong committee" to "present to the next legislature the protest of their State Association against the passage of this bill." We are assured that this committee "will not be idle"; that the "eclectics of the State are thoroughly organized, and, in the event that the dominant school insist upon securing this legislation, will be on hand in force when the legislature convenes."

As a substitute for this bill the eclectics will propose a law requiring the medical colleges of the State to adopt the three-term course, and not admitting any one to practice in the State who cannot present the diploma of a three-term college. That is just exactly what the present bill will require, but evidently that is not enough. A man's fitness for practice is to be judged not by the length of time he has spent in college, but by what he has learned there. The people don't inquire how long a student has attended lectures, and they don't care; but they do want to know if he is competent, and that can be determined only by a personal examination. Moreover, by the proposed substitute the colleges would still grant licenses while conferring diplomas, the very condition which fosters looseness and inefficiency in medical instruction.

Our brethren of the vegetable persuasion will insist further that, if there is to be an examining board at all, there should be one for each school of medicine represented in the State. There are 200 eclectics in Georgia and 25 homeopaths. With only this numerical strength these schools could not maintain efficient examining boards. The strength of the combined boards would be only the strength of the weakest, and such an arrangement would give little protection to the people. After all, there is but one science of medicine. There are differences between the schools on general principles of treatment, but we all have one anatomy, one practice, one surgery and one obstetrics. Wherever these mixed boards are made up of reputable and intelligent practitioners, united upon a common purpose, there is no friction. Mixed boards are now operating smoothly in Minnesota, Iowa, Montana, Missouri, North Dakota and other States. The secretary of one of these says "that they render better service to the public than is done in the few instances where separate boards have been created in compliance with the demands of the several schools of practice. In the States possessing mixed boards that conscientiously perform the duties of a public servant I have yet to hear of any clashing or jealousy among the members thereof."

It will be alleged that this bill is unconstitutional. This old argument, which has done duty in many a weak cause, will be brought out again. But when, in the name of law and morals, did it become unconstitutional for a State to protect the lives and health of its citizens against ignorance and fraud? The general government does not find it unconstitutional to exact the most rigid qualifications of its army, navy and marine surgeons in order that our soldiers and sailors may receive only the best medical attention. It is constitutional for us to establish certain standards of qualification for steamboat pilots and engineers in factories, mills, public and private buildings, etc., in order that the lives and property of people may not be endangered. We require that our lawyers and drug clerks be examined in order that the incompetent may be excluded; nothing unconstitutional about that. We have laws for the prevention of cruelty to animals; there are laws for the protection of our game; the law protects the buzzards of the air, and the terrapins in our mudholes; and all these are regarded as beneficent and constitutional. But when we ask the State to pass a measure which will protect its citizens against illegal and unqualified practitioners of medicine and surgery, a cry is raised by some who appear really to believe it and others who are paid for it that this would be unconstitutional.

The *Journal of the American Medical Association* says: "In view of the important interests committed to the charge of the physician, the necessity that he should possess the necessary qualifications of learning and skill is so great, and his want of them likely to be attended with results so injurious to health and destructive of life, that the power of the State to enact such laws regulating the practice of medicine and surgery as are calculated to exclude and protect the people from ignorant pretenders and charlatans, has been established by repeated adjudications, and is now too firmly settled to admit of doubt."

The "unconstitutional" theory does not work. State after State has rejected it. The Supreme Court of Kansas has ruled (44 Kansas, 565): "The power of the legislature to regulate the practice of medicine, dentistry or surgery is undoubted." The constitutionality of the Nebraska act has just been tested. (*State vs. Wo.*) The

State argued: "We find on comparing this act with similar acts in other States that according to the decisions of those States, and according to the decision of the Supreme Court of the United States, the act is not unconstitutional, but is valid in every particular. It is of the utmost importance that all men lacking in skill, learning and honesty should be excluded from the profession. The tendency of legislation has always been to secure this result. Every effort by the legislature to protect and preserve the lives and health of the people of the State should be looked upon with favor by the courts." The decision of the Supreme Court was an indorsement of this position. When a test case (from Virginia, I think), was taken to the United States Supreme Court, Justice Field ruled (129 U. S. 114): "The power of the State to provide for the general welfare of its people authorizes it to prescribe all such regulations as, in its judgment, will secure, or tend to secure, them against the consequences of ignorance and incapacity, as well as of deception and fraud. . . No one has a right to practice medicine without having the necessary qualifications of learning and skill; and the statute only requires that whoever assumes, by offering to the community his services as a physician, that he possesses such learning and skill, shall present evidence of it by a certificate or license from a body designated by the State to judge of his qualifications." Similar decisions have been rendered by the Supreme Courts of Illinois, Michigan, California, Mississippi, Ohio, Alabama, Iowa and West Virginia.

The establishment of these boards in all parts of the country is no longer a piece of experimental legislation. They are now operating successfully in twenty-four States, one territory, and even among the Cherokee and Choctaw nations. They have demonstrated their usefulness wherever they have been tried, and no State in which the system has been properly conducted has ever abandoned it. In a certain county of one State it is said that no one can be elected to the legislature who is in favor of any interference with the medical practice act. But let us take the evidence :

Dr. Perry H. Millard, St. Paul, Minn., says : "The (present satisfactory conditions) in this State are wholly due to efficient legislation, and the result of the act has been to enhance the welfare of both the profession and the public." Dr. J. M. Hays, ex-Secretary of the North Carolina Medical Society, says in a private letter: "The effect of our medical license act has been exceedingly marked for good. When I say that for eight years about one-third of all applicants for license have been found to be unqualified it is very manifest what the public has escaped. The intelligent public in North Carolina gives our State law its earnest support, and if there has ever been a murmur of dissatisfaction with its operation from any source it has come from some one totally unfit for the practice of medicine, who aspired to obtain license and failed." In Virginia Dr. R. W. Martin, ex-President of the State Medical Society, says: "The law has protected the unsuspecting citizens of the Commonwealth both from the money-getting quackeries of cheats and adventurers, and the injudicious though honest efforts of unqualified doctors. The public recognizes the fact that this law has brought protection, and physicians are frequently congratulated on the improvement that has taken place. The State is almost clear of frauds and impostors. The young men entering the profession have been stimulated to higher and nobler aims. The medical schools are doing better work; they are making greater effort to prepare their graduates to meet the requirements of the law." A private letter from Dr. Michaux, the present Secretary of the Virginia Board, fully corroborates these statements. The New York *Medical Record* (January 28th) testifies that "in those States in which the legal regulation of medical practice has been adopted, good results have followed. The people have been insured to some extent against quackery, and no one's just rights have been encroached upon. The laws have been for the benefit of the people." The *Journal of the American Medical Association* (February 18th) testifies that "wherever such boards have existed they have been of value not only to the State in affording protection to the people from charlatans and unqualified practitioners, but such boards have exercised a most valuable influence in elevating the educational standard of the medical profession."

Let me say in conclusion that this bill is fair, liberal and impartial, and free from reasonable objections. Those who framed it have sought for no favors for themselves or their school of medicine, nor asked for any discriminations against others. The selection of the board is entrusted to the judgment of the governor, with no restrictions except that the different schools are to be represented approximately in proportion to their numerical strength in the State. The bill defines itself as seeking to "protect the people from illegal and unqualified practitioners of medicine." It seeks to secure for the people only such physicians as are competent to practice their profession. That is all the people want, and they are certainly entitled to that much. If Georgia would like to furnish her citizens with this security; if she would like to divorce the right to practice medicine from the empty honor of having a diploma; if she would like to bring about higher standards in her medical schools and stimulate her students to higher aims; if she would like to improve the *personnel* of the medical profession in the State and endeavor to make it what it should be, an intelligent, honest and conscientious body of physicians; then let there be established a Board of Medical Examiners who shall be untrammeled of any college connections, and who shall determine whether a given applicant is qualified to practice medicine in Georgia.

